# Assessing the ability of health information systems in hospitals to support evidence-informed decisions in Kenya

**DOI:** 10.3402/gha.v7.24859

**Published:** 2014-07-31

**Authors:** Elesban Kihuba, David Gathara, Stephen Mwinga, Mercy Mulaku, Rose Kosgei, Wycliffe Mogoa, Rachel Nyamai, Mike English

**Affiliations:** 1 Ministry of Health, Government of Kenya, Nairobi, Kenya; 2 The Health Services, Implementation Research and Clinical Excellence (SIRCLE) Collaboration, Nairobi, Kenya; 3 KEMRI-Wellcome Trust Research Programme, Nairobi, Kenya; 4 Department of Pharmacy and Phamacognosy, School of Pharmacy, University of Nairobi, Nairobi, Kenya; 5 Department of Obstetrics and Gynecology, School of Medicine, University of Nairobi, Nairobi, Kenya; 6 Nuffield Department of Medicine, University of Oxford, Oxford, United Kingdom; 7 Department of Paediatrics, University of Oxford, Oxford, United Kingdom

**Keywords:** health information system, hospital management information system, data quality

## Abstract

**Background:**

Hospital management information systems (HMIS) is a key component of national health information systems (HIS), and actions required of hospital management to support information generation in Kenya are articulated in specific policy documents. We conducted an evaluation of core functions of data generation and reporting within hospitals in Kenya to facilitate interpretation of national reports and to provide guidance on key areas requiring improvement to support data use in decision making.

**Design:**

The survey was a cross-sectional, cluster sample study conducted in 22 hospitals in Kenya. The statistical analysis was descriptive with adjustment for clustering.

**Results:**

Most of the HMIS departments complied with formal guidance to develop departmental plans. However, only a few (3/22) had carried out a data quality audit in the 12 months prior to the survey. On average 3% (range 1–8%) of the total hospital income was allocated to the HMIS departments. About half of the records officer positions were filled and about half (13/22) of hospitals had implemented some form of electronic health record largely focused on improving patient billing and not linked to the district HIS. Completeness of manual patient registers varied, being 90% (95% CI 80.1–99.3%), 75.8% (95% CI 68.7–82.8%), and 58% (95% CI 50.4–65.1%) in maternal child health clinic, maternity, and pediatric wards, respectively. Vital events notification rates were low with 25.7, 42.6, and 71.3% of neonatal deaths, infant deaths, and live births recorded, respectively. Routine hospital reports suggested slight over-reporting of live births and under-reporting of fresh stillbirths and neonatal deaths.

**Conclusions:**

Study findings indicate that the HMIS does not deliver quality data. Significant constraints exist in data quality assurance, supervisory support, data infrastructure in respect to information and communications technology application, human resources, financial resources, and integration.

Health systems strengthening is a global priority (1) and one of the core components is the need to improve health information systems (HIS) (1–3). These are described by the World Health Organization as an *integrated effort to collect, process, report, and use health information and knowledge to influence policy making, program action, and research* (4). In fulfilling this function, a HIS should draw on complete and high-quality data from both population and health facility–based sources (5, 6).

Despite their importance, a number of studies have raised concerns about the performance of HIS suggesting they produce poor-quality data (7–12). In 2007–2008 the Health Metrics Network (HMN) supported a ‘high level’ evaluation in Kenya which raised concerns over the low reporting rates and lack of a policy framework to guide HIS activities (13, 14). These concerns were expressed at a time of increasing demands for performance information, often linked to specific indicators for vertical programs such as malaria or HIV-related mortality rates, maternal mortality, or immunization rates (15). In response, Kenya has invested in policy development (3, 16) and implemented at national scale in September 2011 the computerized District Health Information System version 2 (DHIS2 http://www.dhis2.org/) that is now widely used across Africa.

Within the broader HIS, hospital management information systems (HMIS) is one key component (12), and actions required of hospital management to support data collection and information generation in Kenya are articulated in specific policy documents (3). Responsibilities include vital registration of births and deaths occurring within the facility as part of integrated population-based national reporting systems. In addition, through facility-specific systems, hospitals should report workloads, cause of illness and death by department, illness-specific indicators (for example antenatal HIV testing rates), and overall outcomes stratified by age and sex among other indicators. These hospital related data are collected and reported in Kenya using a paper based subsystem, for primary data collection, with summarized data periodically submitted within an electronic subsystem of DHIS2 for centralized aggregation of data.

Given prior experience suggesting it was sometimes difficult to obtain key hospital data (17, 18), the aim of the study was to evaluate HMIS within hospitals to explore whether efforts to improve HIS have been effective and to inform interpretation of aggregate national reporting.

## Methods

### Indicators

**Fig. 1:**
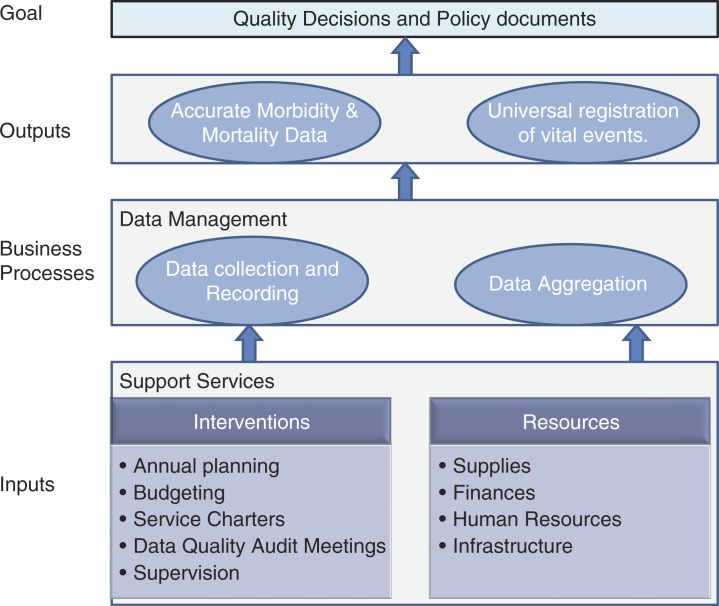
Hospital management information system (HMIS) production framework.

This study focused on HMIS within and serving hospitals. There are few evaluation methods and frameworks to guide comprehensive assessment of HMIS, particularly in a public system setting in a developing country (19). As a result, the study was based on a generic ‘production system’ framework (inputs, processes, and outputs). Choice of indicators was guided by previous work evaluating the national and DHIS (2, 6, 13, 19–22). Key among these tools was the HMN assessment toolkit which was screened to identify a list of potential indicators seeking to address the questions – do HMIS have and organize resources to enable generation of good data; are procedures followed to generate good data; are good data reports produced? – These generic indicators were further refined to fit the hospital setting and a more specific framework was developed (Fig. 1) to examine HMIS in terms of inputs – the resources and institutional support available, business processes – the accuracy of data collection in registers (primary source documents), and outputs – the accuracy of the reported mortality and morbidity figures and vital registration. We further focused on the assessment of processes and outputs within maternal and child health (MCH) clinical areas because of the significance of their respective millennium development goals (MDG http://www.un.org/millenniumgoals/). Appendix 1 lists the specific areas of evaluation linked to these functionally defined HMIS dimensions.

### Survey sites

Kenya has 275 public hospitals that should be able to offer first referral level services as a minimum standard of care. Of these, 40 are ‘internship training centers’ that are typically higher volume public hospitals with: 1) consultants and other technical and supportive staff in each of the four major inpatient disciplines (surgery, internal medicine, obstetrics, and pediatrics), and 2) at least basic laboratory, pharmacy, and radiology services; and at least one working theater. Such centers provide supervised training to trainee clinical staff during a 1-year compulsory internship period prior to full registration. The Ministry of Health was particularly concerned to evaluate performance in these centers expected, by virtue of their resources, to be most likely to represent good practice within the public health system. The resources available were sufficient to survey 22 of these 40 hospitals and sites across Kenya were purposively selected by the Ministry of Health to provide a logistically feasible and geographically representative sample of these hospitals for assessment.

### Data collection

Data reported here were collected by six personnel from The Health Services, Implementation Research and Clinical Excellence (SIRCLE) Collaboration, a technical collaboration between the Ministry of Health, the University of Nairobi, and the KEMRI-Wellcome Trust Research Programme. Data were collected as part of a wider exercise conducted to assess quality of care with at least one SIRCLE team member spending a period of 3–4 days in each of the 22 hospitals. All were experienced health workers themselves and had knowledge of how hospitals are organized structurally, clinically, and administratively. Training was provided to the SIRCLE team including performance of a pilot study. Data collection tools included pre-designed tally sheets, a document review form, and a resource and service organization checklist completed using observations supplemented by directed questions to key informants where necessary.

All the data that were collected were later transcribed using an interface designed in REDCap (Research Electronic Data Capture), a web-based software solution for designing data entry interfaces, by trained data entry clerks. Numeric data were evaluated for range and consistency using built-in checks. Data entry errors or inconsistencies were corrected, where possible, by the principal investigator by referring to the original data collection forms. The clean data set was then exported to STATA version 12 for analysis.

### Statistical analysis

Input indicators, which were mainly related to availability of resources and the status of the institutional arrangements, are summarized across hospitals as a frequency or percentage with a range given, where appropriate, to illustrate the extent of variability. The data were further explored under two categories reflecting hospital size (high and low volume). As there appeared no meaningful difference in results between these hospitals groups, pooled results are presented for all hospitals.

For process indicators, the pooled mean completeness rates (integrity) of the hospital registers (Maternity, Pediatric, and MCH) are reported and the 95% confidence intervals are presented and adjusted for clustering using the *svy* commands in STATA. In the assessment of accuracy of vital notification rates, we compared the number of routinely registered events with survey determined events over the same period (as gold standard) for births and deaths in the MCH inpatient departments. We report the proportion across hospitals of survey confirmed events identified in the routine vital registration system (Appendix 1). To assess the accuracy of hospital reports of mortality (for fresh stillbirths (FSB), neonatal deaths, under 1-year-old deaths (infant deaths)), and live births sent through the DHIS2 database; these were compared with the survey-derived estimates (deemed to be the gold standard) for the same period (Appendix 1). For the live births we also calculated the percentage difference between the two measures for each hospital and used a Bland–Altman plot to illustrate agreement. For output indicators, results presented are for hospitals that had the relevant source documents.

### Ethics

Scientific and ethical approval for the study was obtained from the Kenya Medical Research Institute National Scientific and Ethical Review Boards. The Ministry of Health also approved the study and the study was explained to hospital management teams who provided their assent prior to data collection.

## Results

### Inputs

#### Institutional interventions

In the domain of institutional interventions the majority of the hospitals (21/22, 95%) had developed an annual operational plan (AOP) for the HMIS department with clearly defined performance objectives. Similarly most HMIS departments (18/22, 82%) were actively involved in efforts to develop a costing and budgeting approach for departmental activities. HMIS departments’ service charters were found in 16/22 (73%) of the hospitals but these were complete as stipulated by the Ministry of Health guidelines in 13/22 (59%). However, very few HMIS departments (3/22, 14%) had carried out a data quality audit meeting or established a data quality committee in the 12 months prior to the survey. Most HIS departments (19/22, 86%) had received one supervisory visit in the 12 months prior to the survey.

#### Resources

Hospitals have traditionally relied for supply of formal registers and summary forms on national government. We observed immunization registers and the immunization and nutrition summary forms to be universally available but, of the remaining eight mandatory forms and registers, each was missing in between 1 and 6 of 22 hospitals. At hospital level, HMIS departments were generally poorly financed. On average 3% of the total annual income, from cost sharing and government grants, was allocated to the HMIS departments with a range of 1–8% compared with a policy requiring that at least 10% should be allocated to information services. There was a large deficit in staffing levels at the HMIS departments. Across all hospitals only 47% (111/233) of the positions for records officers recommended by the government in workforce norms were filled, with non-governmental organizations employing 14/111 (12%) of these existing staff. Within hospitals between 1/9 and 17/21 records officer posts were unfilled. Lack of investment in records staff was further apparent in limited efforts to provide training; only 12/22 (54%) hospitals had any training plan related to data management and in 7/12 (58%) of these the training plan was not adequately financed.

All the hospitals had functional computers (varying from 7 to 111 per hospital); 17/22 (77%) hospitals had functional local area network (LAN); and all had some internet connectivity. Aggregated across the 16 hospitals that had complete data 340/785 (43.3%) of the computers had an internet connection (range per hospital 4/111, 3.6%, to 50/55, 91%). The information technology support and maintenance of the system was predominantly handled by external contracting companies in 13/22 (59%) hospitals, by internal information technology staff in only 7/22 (31%) hospitals, and one hospital did not have a formal arrangement for these services.

The DHIS2 (23) had been introduced in all hospitals but only 15/22 hospitals were making use of its analysis and presentation functions. None of the MCH departments’ in charges (*n =* 66) responsible for data management in their dockets had been trained on how to operate the DHIS2. In addition, of the 176 senior managers in the hospitals visited, only 35 (19.8%) had access rights to the DHIS2 hospital reporting system.

Separate to DHIS2, just more than half of hospitals (13/22, 59%) had implemented additional electronic health records management information systems. Our observations indicated that the dominant function of these automated systems was to improve financial (administrative) management, particularly patient billing for diagnostic services or treatments and subsequent accounting, and they were predominantly located and operational in the outpatient departments. Only 7/13 (54%) of these automated systems could produce reports that were valuable for preparation of routine departmental DHIS2 reports.

### Business processes

#### Completeness

The integrity of the registers was classified as good (completeness rate of >90%), moderate (completeness rate of 70–90%), and poor (completeness rate of <70%) based on a scoring system adopted from National Health Service, UK. Completeness of required data in the MCH register was 90% (95% CI 80.6–99.3%) averaged across hospitals with a range of 80–100%. This was somewhat higher than the inpatient maternity department, with a mean completeness rate of 75.8% (95% CI 68.7–82.8%, a range of 22–88.8%) and substantially higher than for the inpatient pediatric register, mean completeness of 58% (95% CI 50.4–65.1%, range 1–80.7%). Furthermore our observations showed that patients’ demographic data were typically collected whereas data on patients’ outcomes and management were most likely to be missing.

### Outputs

#### Vital events notification rates

The notification of deaths to the registrar of deaths and births was poor in the departments assessed. Indeed the survey team was unable to collect data on under 1-year-old deaths (infant deaths) in five hospitals because of unavailability of source documents; the birth and death notification forms. Where available, observations indicated that registration was mostly done after the relatives had paid hospital charges (Appendix 2). Of the 157 FSB that occurred in May 2012 across the 11 hospitals with data, only 33 (21%) had been registered as vital events. Similarly, of the 170 neonatal deaths that occurred in that month across 18 hospitals only 25.7% (52/170, 95% CI 22.5–28.9%) had been registered. As the neonatal deaths contributed about 61% (170/276) of all under 1-year-old deaths in these hospitals, the overall notification rate for deaths under 1-year-old was also low, at only 42.6% (140/276, 95% CI 24.8–60.4%) of survey identified events. The registration of live births was somewhat higher than the other vital events. Of the 5,961 live births that occurred in May 2012 across 19 hospitals, 71.3% (4,399/5,961, 95% CI 69.1–73.5%) had been registered.

#### Accuracy of reported events

**Fig. 2:**
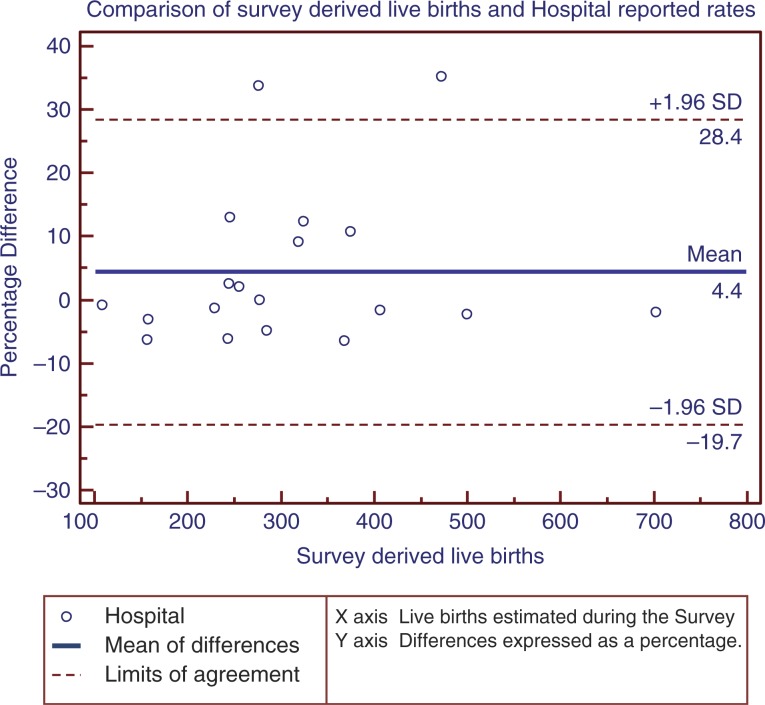
The percentage difference between hospital reported and survey rates for live births for the month May 2012.

The poor state of the original source documents and unavailability of hospital reports made it difficult to assess all the 22 sites. We report findings for three indicators – live births, FSB, and neonatal deaths (Appendix 2). There appeared to be over-reporting of live births across the 19 hospitals with data (6,257/5,474), survey mean of 104.2% (95% CI 98.4–110.2%). Overall there was marked variability in the percentage differences between the hospital reported rates and the survey rates for live births (Fig. 2). On the contrary the hospital reported rates for FSB were lower than the gold standard (123/157), survey mean of 68.9% (95% CI 63.4–74.4%). Similarly there was under-reporting of neonatal deaths. Of the 170 neonatal deaths that had taken place across 17 hospitals, 162 had been reported with a mean reporting rate of 71.8% (95% CI 65.1–78.6%).

## Discussion

In recent times the need for good information for planning and decision making has gained prominence (6, 24).The utility of health care utilization data aggregated by the national or county HIS depends on the quality of primary data collection and reporting systems (HMIS) at the health facility level (25–27). Study findings indicate this process at hospital level is likely to be undermined by inadequate inputs if these are compared with existing policy guidance. Hospitals were often compliant with requirements for ‘one off’ exercises such as preparation of annual plans, departments’ performance objectives, and budgets related to HMIS probably because of the high importance attached to these exercises by the Ministry. However the financial resources allocated to HMIS were low and fell short of the recommended levels (3), with frequent stock outs of stationery and mandatory reporting templates, and inadequate human resources. Task shifting seemed to be the main strategy used by hospitals in a bid to address the deficits in records officers with nurses taking a leading role in data collection and compilation (12) and employment of temporary records staff on short-term contracts.

Application of information technology may greatly facilitate data management activities and improve the quality of data (6, 28–30), and hospitals in Kenya are investing in this technology. They are often doing this in the absence of local, professional technical support; however, this investment largely focuses on administrative management, notably improving revenue collection. Recognition that such systems might produce important health information was often lacking, indeed about half of the HMIS software implemented by hospitals lacked the capacity to produce health related reports in a format that is prescribed by the government, and there were no instances where local HMIS systems were linked to DHIS2, the national health information reporting architecture. Perhaps contributing to this, most of the hospital managers were likely unaware of wider health information needs, they were not trained how to use DHIS2, and indeed they had not even opened a user account.

Good quality information, whether generated by manual or technological systems, can support quality decision making which ultimately leads to improved quality of care (27). Conversely, inaccurate information can lead to poor choices in health investments. Our study findings were consistent with other studies in developing countries that suggest that routinely reported data were of poor quality (7–12). Official hospital reports were often not representative of utilization levels or aggregate patient outcomes. Of particular concern were poor compliance with roles of the hospitals in contributing to national vital registration of births and deaths and likely inaccurate reporting of MCH events. Such a situation was likely exacerbated by staffing inadequacies and three additional factors. These factors are the absence of data quality assurance mechanisms in practice with little attention paid to this issue even in policy (14, 31, 32), infrequent supervisory support that might correct faults and weaknesses in the HMIS departments (33), and organizational barriers to registration of events. For example registration of deaths and births is one of the last steps in a sequence of procedures required to formally discharge a patient, mainly done after hospital fees are paid. As noted in another study this conditionality can reduce the focus on registration among the health workers and families (34).

Limitations of this study are the non-random sampling of study sites which limits our ability to generalize the results more widely. The assessment of data quality concentrated on specific departments dealing with pediatric and maternal health. Given the focus of local policy on their respective MDGs, these departments are likely to be well resourced and we might expect better performance in data collection in pediatric and maternal health departments. Other limitations reflected inability to access source documents in some hospitals. Their absence is perhaps a reflection of considerable challenges in managing information and so our results, based on available documents, may in fact be an over optimistic view of information quality. In addition this study was not designed to assess the association and interactions between human factors, availability of resources, and accuracies of the reported estimates; a topic that should be the subject of future research.

## Conclusions

HMIS are faced by significant constraints including: inadequate financial and human resources; limited attention paid to data quality assurance and supervisory support; and failure to maximize the potential of emerging information technologies. The inadequate operational capacity at the hospital suggests that current efforts at the national HIS level have not percolated through the system as witnessed by the HMIS suboptimal performance. As noted in other studies unreliable data is associated with low demand for data (30, 35) which ultimately result in low investment in these systems. To improve the utility of the nationally reported rates there is need to focus on the capacity and performance of HMIS.

## Appendix

**Table:** Appendix 1: HMIS dimensions and calculation of selected indicators

System level	Dimensions	Indicator	Data collection and indicator calculations
Outputs	Quality of data	Accuracy of the admissions and mortality rates reported by hospitals as core components of monitoring maternal and child health	In each inpatient department (maternity, pediatric, or neonatal), patient episode entries in the registers were reviewed and the number of admissions and corresponding outcomes at discharge tallied for the month of May 2012 to arrive at survey-derived rates for live births, fresh stillbirths (FSB), neonatal and infants deaths for each hospital. These were considered to be the gold standard and contrasted with routinely reported hospital data for the same month.
	Birth and death registration	Proportion of vital events registered	Using the registration forms as source documents, a tally was done for live births and deaths (fresh stillbirths, neonatal, under 1 [infants], and under 5 years) within the facility notified in the month of June 2012 across all hospitals. The tally was compared with corresponding survey-derived rates.
Processes	Data collection and analysis	Integrity of register data collection and analysis in maternity, pediatric, and maternal child health clinic	50 patient episode entries for each of the maternity wards (*n =* 31 fields), pediatric wards (*n =* 14 fields), and maternal child health clinics (*n =* 11 fields) were randomly selected from 1 April 2012 to 30 June 2012 and reviewed for each hospital. Valid data elements tallied to allow calculation of a proportion representing completeness of data for maternity, pediatric, or MCH registers (calculated as tally of total valid entries/50*31, 14 or 11 for maternity, pediatric, and MCH locations, respectively).
	Institutional arrangements	Existence of institutional interventions that allow optimal performance of the HMIS	Interventions such as data quality meetings, supervision visits, annual operation planning, and budgeting put in place were examined by reviewing facility administration records (committee meeting minutes, visitors book, and reports)
	Supplies	Availability of stationery in the HMIS office and inpatient departments	Data on availability of supplies such as printing papers, files, pens, formal registers, and summary forms were collected during a facility walk through
Inputs	Financial resources	Percentage of hospital total annual financial resources allocated to HMIS department	Financial allocation to HMIS and total annual budget appropriated at the hospital was determined by reviewing hospital annual financial statements and reports for 2011–2012 financial year.
	Human resources	Percentage fill rate of the recommended records officers positions	Data on number of records officers posted in each hospital were extracted from the personnel records and reports and compared with recommended records officer positions
	Infrastructure	Existence of a robust data infrastructure	Data on availability of information and communication technology equipment were collected using a facility walk through and through direct observations.

**Table:** Appendix 2: Summary of results tables

System level	Dimensions	Indicator	Survey score (95% CI where applicable)
	Consistency of hospital reported rate with	Live birth	104.3 (98.4–110.2%)
	survey-derived estimate	Fresh stillbirths (FSB)	68.9 (63.4–74.4%)
		Neonatal death	71.8 (65.1–78.6%)
	Birth and death registration	Proportion of live births registered	71.3 (69.1–73.5%)
		Proportion of registered FSB	21%
Outputs		Proportion of registered neonatal death	25.7 (22.5–28.9%)
		Proportion of registered infant death	42.6 (24.8–60.4%)
		Integrity of inpatient maternity register maternity	75.8 (68.7–82.8%)
Processes	Data collection and analysis	Integrity of inpatient pediatric register	58 (50.4–65.1%)
		Integrity of MCH register	90 (80.6–99.3%)
	Institutional arrangements	HMIS departments AOP	21/22, 95%
		HMIS participation in budgeting	18/22, 82%
		Data quality audit	3/22, 14%
	Financial resources	Percentage of hospital total annual financial resources allocated to HMIS department	3%
Inputs	Human resources	Percentage fill rate of the recommended records officers positions	47%
	Infrastructure	Availability of a functional LAN	17/22, 77%
		Internet connectivity	22/22, 100%
		Automation of HMIS activities	13/22, 59%
